# Strong horizontal and vertical connectivity in the coral *Pocillopora verrucosa* from Ludao, Taiwan, a small oceanic island

**DOI:** 10.1371/journal.pone.0258181

**Published:** 2021-10-11

**Authors:** Stéphane de Palmas, Derek Soto, Ming-Jay Ho, Vianney Denis, Chaolun Allen Chen

**Affiliations:** 1 Biodiversity Research Center, Academia Sinica, Taipei, Taiwan; 2 Department of Life Science, National Taiwan Normal University, Taipei, Taiwan; 3 Biodiversity Program, Taiwan International Graduate Program, Academia Sinica and National Taiwan Normal University, Taipei, Taiwan; 4 Institute of Oceanography, National Taiwan University, Taipei, Taiwan; 5 Green Island Marine Research Station, Marine Science Thematic Centre, Biodiversity Research Center, Academia Sinica, Green Island, Taitung, Taiwan; 6 Department of Life Sciences, Tunghai University, Taichung, Taiwan; National Cheng Kung University, TAIWAN

## Abstract

Mesophotic habitats could be sheltered from natural and anthropogenic disturbances and act as reproductive refuges, providing propagules to replenish shallower populations. Molecular markers can be used as proxies evaluating the connectivity and inferring population structure and larval dispersal. This study characterizes population structure as well as horizontal and vertical genetic connectivity of the broadcasting coral *Pocillopora verrucosa* from Ludao, a small oceanic island off the eastern coast of Taiwan. We genotyped 75 *P*. *verrucosa* specimens from three sites (Gongguan, Dabaisha, and Guiwan) at three depth ranges (Shallow: 7–15 m, Mid-depth: 23–30 m, and Deep: 38–45 m), spanning shallow to upper mesophotic coral reefs, with eight microsatellite markers. F-statistics showed a moderate differentiation (F_ST_ = 0.106, p<0.05) between two adjacent locations (Dabaisha 23–30 and Dabaisha 38–45 m), but no differentiation elsewhere, suggesting high levels of connectivity among sites and depths. STRUCTURE analysis showed no genetic clustering among sites or depths, indicating that all *Pocillopora* individuals could be drawn from a single panmictic population. Simulations of recent migration assigned 30 individuals (40%) to a different location from where they were collected. Among them, 1/3 were assigned to deeper locations, 1/3 to shallower populations and 1/3 were assigned to the right depth but a different site. These results suggest high levels of vertical and horizontal connectivity, which could enhance the recovery of *P*. *verrucosa* following disturbances around Ludao, a feature that agrees with demographic studies portraying this species as an opportunistic scleractinian.

## Introduction

Mesophotic Coral Ecosystems (MCEs) are deeper sections of shallow tropical and subtropical coral reefs [1]. Generally located from 30 m to as deep as 150 m [2], MCEs can be sheltered from warm-water bleaching, tropical storms, and anthropogenic disturbances [3–5]. In parallel, MCEs may act as a reproductive refuge for some reefal organisms, and supply recruits to shallow water reefs following major disturbances [6–8], thus maintaining species richness and community structure, and facilitating recovery after disturbances [9–11]. However, the role of MCEs as ecological refuges, formalized as the “Deep Reef Refuge Hypothesis” (DRRH [7, 8]), depends on the frequency and intensity of depth-dependent disturbances, the local ocean dynamics, and the taxon considered—specifically that taxon’s depth distribution range and reproductive traits [5, 12–15]. Particular emphasis is now given to identifying locations and taxa that, within the DRRH framework, may persist facing major disturbances [16–27] or not [28–32].

In most benthic marine species, recruitment and settlement of new individuals are achieved through pelagic larval movement [9]. While larval dispersal and the effective recruitment at shallow and mesophotic depths are critical to the reef dynamic [33], quantifying them remains a challenge [34] considering that benthic larvae have limited mobility and small recruits. Although biophysical models predict that such movements across depth significantly contribute to demography and resilience in reef coral populations [15], genetic connectivity approaches offer a valuable proxy for assessing population structure and estimating larval flux across depths [6, 12, 35–45]. To date, genetic approaches have revealed contrasting patterns of connectivity between shallow and deep populations of reef corals, suggesting that connectivity is taxon- and location-specific [1, 46]. However, despite a growing body of literature on this topic, research evaluating connectivity between shallow and mesophotic coral populations is still considered to be preliminary [1, 47].

In Taiwan, well-developed fringing reefs have been observed around Ludao, a small volcanic island 33 km off the coast of Taitung. Along the bathymetric gradient, *Pocillopora verrucosa* is one of the most dominant scleractinian hard corals, distributed from the near-surface to as deep as 55 m [48]. Despite variation in corallum micro and macro morphologies with depth [49], sequencing of the mitochondrial Open Reading Frame marker (mtORF, from a gene with unknown function) has confirmed this species’ distribution across a large bathymetric gradient in Ludao [50]. Several microsatellite markers (also called Simple Sequence Repeats, SSRs) have been developed for *Pocillopora* species [51–54] and were used to investigate species diversity and population connectivity across their biogeographic distribution [55–58]. However, to date little has been done to assess connectivity across depths, population structures, or larval fluxes between shallow and deep populations of *Pocillopora verrucosa*. The present study aims to make these assessments.

## Materials and methods

### Sampling and DNA purification

**Fig 1:**
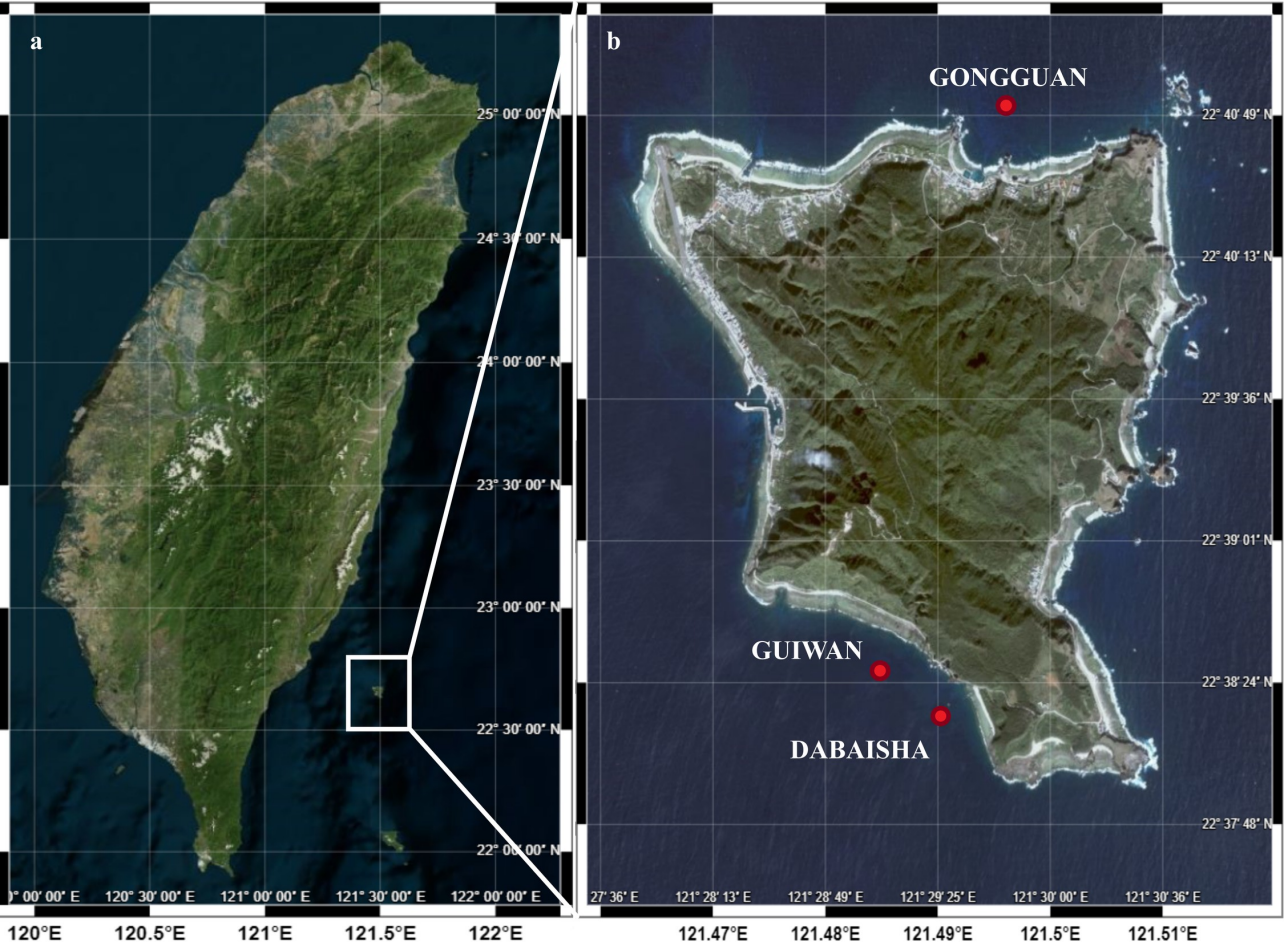
Ludao, Taiwan; and sampling sites. **a.** Ludao; **b.** Details of the island with position of the sampling sites Gongguan, Guiwan, and Dabaisha. Source: Ocean Data Bank, National Taiwan University (http://www.odb.ntu.edu.tw/).

In 2016 and 2017, *Pocillopora verrucosa* specimens were collected at three sites around Ludao—Gongguan, (22.683530°N, 121.496631°E), Guiwan, (22.639212°N, 121.483529°E), and Dabaisha (22.637706°N, 121.490844°E)—using compressed air or Trimix diving, with a minimum distance of 3 m between samples to avoid collecting colonies that might be clones (Fig 1). A collection permit was obtained from the Taitung County Government (1040000285) and all specimens were registered at the Biodiversity Research Museum (Academia Sinica, Taiwan). Distances between sites are < 6 km (Fig 1).

Eighty-five colonies originating from three depth ranges—Shallow (7–15 m, hereafter “S”), Mid-depth (23–30 m, hereafter “M”), and Deep (38–45 m, hereafter “D”)—were previously confirmed to be *Pocillopora verrucosa* by mtORF sequencing [50]. Nine locations were defined accordingly: Gongguan S, Gongguan M, Gongguan D, Guiwan S, Guiwan M, Guiwan D, Dabaisha S, Dabaisha M, and Dabaisha D. Locations are separated by vertical distances (i.e., depth) and horizontal distances (i.e., perpendicular to the depth axis) so that the frontal distances between locations from the same site are < 100 m.

DNA extraction process followed De Palmas et al. [50]. Additionally, DNA extracts were purified and concentrated using a sodium acetate (3M, pH 5) precipitation method. For each DNA sample, 1/10 volume of sodium acetate was added, and mixtures were gently inverted for 10 min. Three volumes of pure ethanol were added, and solutions were kept at -20°C overnight. Samples were centrifuged at 14,000 g for 30 minutes and DNA pellets were rinsed three times with -20°C ethanol. The DNA pellets were re-suspended in 30 μL TE buffer (1X, USB Corporation, Cleveland, OH, USA). DNA concentration and quality were measured on a Nanodrop 2000 (Thermo Fisher Scientific) and diluted aliquots were prepared to homogenize the DNA concentration of each sample before Polymerase Chain Reaction (PCR).

### Microsatellite marker selection

Microsatellite marker effectiveness was determined by amplifying eight samples haphazardly picked with 30 microsatellite markers, accounting for most microsatellites developed for *Pocillopora* species. Each PCR mixture contained 15 μL of Master Mix RED (Ampliqon, Odense M, Denmark), 15 μL of ddH2O, 2 μL of each pair of primers (2.5 μM), and ~50 ng of DNA template. PCR conditions were as follows: 60 s of denaturation step at 94°C; 40 cycles of 60 s at 94°C, 60 s at the annealing temperature (Ta), and 60 s at 72°C; and an extension step at 72°C for 5 min. PCR amplifications were checked on a 2% agar gel to confirm amplification success rate and product size. Successful amplifications were diluted 22X before processing on a 5400 Fragment Analyzer™ Automated CE System (Agilent, CA, USA) using a dsDNA 905 reagent kit (1 to 500 bp). Any markers displaying low PCR amplification success or non-specific PCR amplification (multiple additional PCR products) were removed from subsequent analyses.

### Microsatellite screening and allele scoring

Loci that passed the selection process were amplified to genotype the 85 *P*. *verrucosa*. Each PCR product was run three times on the fragment analyzer (as described above) to account for the accuracy of the fragment analyzer (i.e., 2–3 bp, per the manufacturer’s instructions). The three repeated electropherograms were scored using Prosize 3.0 (Agilent, CA, USA) and allele repeat size was then estimated using GenAlEx 6.5 [59] based on the three scored electropherograms. The estimation of repeat size and repeated allele scoring were used to solve allele scoring ambiguity. Micro-Checker 2.2.3 [60] was used to detect and correct eventual scoring inconsistencies and scoring errors due to stuttering and large allele drop-out. Samples showing low standard scoring quality were removed from the analysis.

### Computational analysis

Arlequin 3.5.2.2 [61] was used to calculate the total number of alleles (Na), the observed (Ho) and expected (He) heterozygosities, and the exact probability of departure from Hardy-Weinberg equilibrium for each locus (pHWE) with 10^6^ steps in a Markov chain and 10^5^ dememorization steps. Arlequin was also used to investigate linkage disequilibrium between all pairs of loci with 5 x 10^5^ permutations. Null allele frequency was inferred using MicroChecker 2.2.3 [60] with a 95% confidence interval and 10^5^ randomizations.

Genetic differentiation among all locations was estimated using a pairwise F_ST_ comparison at all loci and their statistical significance with 5 x 10^5^ permutations in Arlequin. Pairwise F_ST_ were also calculated using FreeNa software [62] with the number of replicates fixed at 50,000. Unlike Arlequin, FreeNa takes into consideration the presence of null alleles and provides an accurate estimation of F_ST_ values [62]. To account for the low number of samples per location, pairwise F_ST_ were also computed at the site level (pooling 3 depth ranges at one site together) and at the depth level (pooling similar depth ranges from all 3 sites together).

Genetic structuring was assessed using STRUCTURE 2.3.4 [63] with 10^6^ Markov Chain Monte Carlo simulations, a burn-in period of 10^5^ and three iterations for each K from k = 1 to k = 12. The LOCPRIOR option was used (to account for the location characteristics) under the admixture model and assuming independent allele frequency. The web-based program STRUCTURE HARVESTER 0.6.94 [64] was used to estimate the K number of populations and compare them to the web-based program CLUMPAK [65]. A principal coordinate analysis (PCoA), which summarizes genetic distances between individual multi-locus genotypes to cluster individuals/locations relative to each other, was performed at the location level using GenAlEx 6.5 [59].

In order to detect individuals with a recent migration history, we use the “Detection of first-generation migrants” option from Geneclass 2 [66] which assigns potential new migrant to their most likely source population [67]. This option calculates the likelihood L_home_/L_max_ (with L_home_ the likelihood computed from the population where the individual was sampled and L_max_ the highest likelihood value among all population including the population where the individual was sampled) using the frequencies-based method from Paetkau et al. [68] and Monte-Carlo resampling algorithm with 10,000 simulated individuals and a type I error threshold α = 0.05 [67]. To account for the low number of samples per locations, this simulation was also computed at the site (pooling 3 depth ranges from one site together) and at the depth level (pooling similar depth range from all 3 sites together).

## Results

Of 85 *P*. *verrucosa* specimens genotyped in this study, 75 produced satisfactory amplification and scoring results (≤ 4% of data missing) for eight microsatellite markers, *Psp_16*, *Psp_23*, *Psp_29*, *Psp_32*, *Psp_35*, *Psp_39*, *Psp_41*, and *Psp_48* (Table 1). Full dataset with allele scoring, mtORF haplotype and museum numbers is provided in S1 Table.

**Table 1:** Characteristics of microsatellite markers tested in this study. Initial PCR amplification success was evaluated for a subset of eight samples. Loci displaying PCR amplification success >75% and expected fragments size for the initial subset (**in bold**) were used to genotype the 85 *Pocillopora verrucosa* or otherwise excluded from this study.

Markers	Forward primer sequence (5ʹ–3ʹ)	Reverse primer sequence (5ʹ–3ʹ)	Ta (°C)	References	PCR success (N = 8)	Scored fragment status	PCR success (N = 85)	Fragment scoring status
Pd2-001	CAGACTTGTCGGAATGAAAGC	TTTTGTTTATAAGTCGATACAATGCA	55	Starger et al. 2008	<25%	-	-	-
Pd3-002	ATCCGAATACAAGCGAAACG	CAAAGCTTCTATCAGAAAATGCAA	55	Starger et al. 2008	>50%	expected fragments size	<50%	-
Pd2-003	CCTCTTCCTGTTTGGGCTCT	TCTGCATTACGTTTGTTTGACA	55	Starger et al. 2008	>50%	major additional products	<50%	-
Pd3-004	ACCAGACAGAAACACGCACA	GCAATGTGTAACAGAGGTGGAA	55	Starger et al. 2008	>50%	major additional products	-	-
Pd3-005	AGAGTGTGGACAGCGAGGAT	GTTCCTTCGCCTTCGATTTT	55	Starger et al. 2008	>50%	major additional products	-	-
Pd2-006	ATCTCCATGTGATCGGCATT	GTTCCCCCAGCTGAGAAGTT	55	Starger et al. 2008	>50%	expected fragments size	<50%	-
Pd2-007	AAGAAGGTGTGGTATTTCAGAGGG	GGTGGATAAAGTATTTCTCACTCTTGG	60	Starger et al. 2008	>50%	expected fragments size	<50%	-
Pd3-008	AGTTGAGGTTGTTGAAACATG	TCCATGCAGAACCCC	60	Starger et al. 2008	>50%	major additional products	-	-
Pd3-009	CCAATGCGTCCGTAGCTCTC	ATCACCTAAAAATTTCAGTCCCTTACC	47	Starger et al. 2008	<25%	major additional products	-	-
Pd3-010	CTGATCAACAAACTGGGAGGC	TCATTAGAAATCATCTTGATTTGATAAGG	47	Starger et al. 2008	no amplification	-	-	-
Pd11	TCGTTTGAAGGGAAATGCTC	GCATGCTATGTATGCGAGA	60	Torda et al. 2013	>50%	low specificity	<50%	-
Pd13	TGTTCCTCTCTTTCTCTCTTCCA	CATTTATGTTCCTTTCACGGC	60	Torda et al. 2013	>50%	major additional products	-	-
PV2	CCAGGACCCATTTATACTCC	TGCAGTGTTCTACTTGTCAGTGC	56	Magalon et al. 2004	<25%	-	-	-
PV5	GTCATCACGCAAAGTTCC	GAATAGCCTGCGTTTATTTGG	56	Magalon et al. 2004	<25%	-	-	-
PV6	CTTTCCCGACCAGTTTAGGG	AGCCGTTCAGCTACCTATGG	56	Magalon et al. 2004	<25%	-	-	-
PV7	GAGATGGATGGAGACTGC	GGTATCTCTGTGCTCAGTTCTTTG	55	Magalon et al. 2004	no amplification	-	-	-
Poc40	TTATTATATGGGTGTATGC	CTCAAAGTGCGATTAAAGCC	55	Pinzón & Lajeunesse 2011	no amplification	-	-	-
Psp_01	TCGTTCAATCCACTGACTGC	CCTTTGGATGCGATGTAAT	56	Nakajima et al. 2017	<25%	major additional products	-	-
Psp_02	CTGTGCTGGAATTCCCCTTA	AGCCTACGGCGCAATAGTAG	56	Nakajima et al. 2017	<25%	major additional products	-	-
Psp_10	AGGCGAAGCCATAATGTTGT	CTTCGTTGTGGGCTAAGAGG	56	Nakajima et al. 2017	no amplification	-	-	-
**Psp_16**	**CCCGCTGCTGAGTAAGAATC**	**AGAGAAACTGCAAAACCGC**	**56**	**Nakajima et al. 2017**	**>75%**	**expected fragments size**	**>95%**	**expected fragment size**
Psp_18	CACACGTTTTATGACAACGGA	ATAAGCCGTAGGCCCTGTCT	56	Nakajima et al. 2017	<25%	-	-	-
**Psp_23**	**ACCATTGCCATCACTGTTCA**	**TTCATTCATTCGTATTGGCG**	**56**	**Nakajima et al. 2017**	**>75%**	**expected fragments size**	**>95%**	**expected fragment size**
**Psp_29**	**TTTCGTACCAAAATCCAGGC**	**TTTTTCAGTCGCAAGAGGC**	**56**	**Nakajima et al. 2017**	**>75%**	**expected fragments size**	**>95%**	**expected fragment size**
**Psp_32**	**AAGCACGCAATTCAGCCTAT**	**AGCCTAAGACGAATCGAGCA**	**56**	**Nakajima et al. 2017**	**>75%**	**expected fragments size**	**>95%**	**expected fragment size**
Psp_33	CCATTTCCCGAATCTCTCTC	CTCGTCGCCCAGATATAAA	56	Nakajima et al. 2017	>75%	expected fragments size	>95%	major additional products
**Psp_35**	**TGGCTGATGTCTGTGGGTAA**	**CGCGATTATCGAAAGTTTG**	**56**	**Nakajima et al. 2017**	**>75%**	**expected fragments size**	**>95%**	**expected fragment size**
**Psp_39**	**TCTTTACAGCACAGGAGCCA**	**TTTTTCTTGCGGTCCAATTC**	**56**	**Nakajima et al. 2017**	**>75%**	**expected fragments size**	**>95%**	**expected fragment size**
**Psp_41**	**CGCACAAGGAAAATTTGTT**	**TTCCACACCAGAAGATGACG**	**56**	**Nakajima et al. 2017**	**>75%**	**expected fragments size**	**>95%**	**expected fragment size**
**Psp_48**	**TGTAAATTCAAGAGAATGGGCA**	**GTTTCCTGATGGTGTTCT**	**56**	**Nakajima et al. 2017**	**>75%**	**expected fragments size**	**>95%**	**expected fragment size**

Loci were polymorphic, but not for all combinations of location and marker (48 combinations in total): *Psp_29* was monomorphic at Dabaisha D and Gongguan S; *Psp_35* was monomorphic at Dabaisha M, Gongguan S, and Gongguan M; and *Psp_39* was monomorphic at Gongguan M (Table 2). The number of alleles per location and locus ranged from one to 19 (Table 2). Twelve combinations of location and loci deviated from Hardy-Weinberg equilibrium (Table 2), representing 16% of the total combinations. Linkage disequilibrium was found in *Psp_29*/*Psp_39*, and *Psp_32*/*Psp_48*. Null alleles were detected in *Psp_29*, *Psp_35*, *Psp_39*, *Psp_41*, and *Psp_48*. No clone was detected.

**Table 2:** Summary of genetic diversity statistics across loci by location. N: number of samples, n: number of specimens which produced data at each locus, N_a_: number of alleles, H_o_: observed heterozygosity, He: expected heterozygosity, pHWE: p-value for exact tests of Hardy–Weinberg equilibrium (significant values are in bold, ns for non-significant,—indicates the presence of monomorphic loci).

Site	Statistic	Psp_16	Psp_23	Psp_29	Psp_32	Psp_35	Psp_39	Psp_48	Psp_41
Dabaisha S N = 14	n	14	14	12	14	14	14	11	13
	N_a_	4	7	2	19	4	2	8	8
	H_o_	0.714	0.642	0.083	1.000	0.071	0.357	0.727	0.692
	H_e_	0.695	0.767	0.228	0.965	0.325	0.452	0.800	0.775
	pHWE	**<0.05**	ns	ns	ns	**<0.05**	ns	ns	ns
Dabaisha M N = 6	n	6	5	5	6	6	5	5	6
	N_a_	4	6	2	10	1	2	5	4
	H_o_	0.166	0.600	0.200	1.000	**-**	0.200	1.000	0.333
	H_e_	0.712	0.866	0.200	0.969	**-**	0.200	0.755	0.772
	pHWE	**<0.05**	ns	ns	ns	**-**	ns	ns	ns
Dabaisha D N = 9	n	9	8	7	9	9	9	9	8
	N_a_	3	5	1	15	3	2	7	6
	H_o_	0.666	0.750	**-**	0.888	0.111	0.222	0.666	0.625
	H_e_	0.568	0.783	**-**	0.973	0.307	0.366	0.784	0.808
	pHWE	ns	ns	**-**	ns	ns	ns	ns	ns
Gongguan S N = 5	n	5	5	5	5	5	5	5	5
	N_a_	4	4	1	9	1	2	5	5
	H_o_	1.000	0.800	**-**	1.000	**-**	0.200	0.800	0.200
	H_e_	0.777	0.711	**-**	0.955	**-**	0.200	0.822	0.866
	pHWE	ns	ns	**-**	ns	**-**	ns	ns	**<0.05**
Gongguan M N = 7	n	7	7	7	7	7	7	7	7
	N_a_	4	6	2	11	1	1	8	8
	H_o_	0.714	0.428	0.428	0.857	**-**	**-**	0.714	0.714
	H_e_	0.714	0.835	0.362	0.967	**-**	**-**	0.868	0.901
	pHWE	ns	**<0.05**	ns	ns	**-**	**-**	ns	**<0.05**
Gongguan D N = 10	n	10	10	9	10	10	10	10	10
	N_a_	4	6	2	12	3	3	6	7
	H_o_	0.500	0.800	0.111	0.800	0.200	0.400	0.400	0.700
	H_e_	0.647	0.747	0.111	0.942	0.194	0.352	0.821	0.831
	pHWE	**<0.05**	ns	ns	**<0.05**	ns	ns	ns	ns
Guiwan S N = 7	n	7	7	7	7	7	5	3	7
	N_a_	5	6	3	10	2	2	3	3
	H_o_	0.857	0.714	0.142	0.571	0.000	0.200	0.666	0.000
	H_e_	0.824	0.791	0.384	0.956	0.263	0.466	0.600	0.615
	pHWE	ns	ns	ns	**<0.01**	ns	ns	ns	**<0.01**
Guiwan M N = 7	n	7	7	7	7	7	6	6	6
	N_a_	5	5	2	10	3	2	4	7
	H_o_	0.714	0.571	0.142	0.857	0.142	0.000	0.500	0.666
	H_e_	0.769	0.758	0.142	0.978	0.274	0.484	0.636	0.909
	pHWE	ns	ns	ns	ns	ns	**<0.05**	ns	ns
Guiwan D N = 10	n	10	10	10	10	9	10	9	10
	N_a_	4	6	3	13	2	2	7	6
	H_o_	0.500	0.700	0.200	1.000	0.000	0.400	0.555	0.200
	H_e_	0.726	0.726	0.357	0.963	0.209	0.336	0.771	0.747
	pHWE	ns	ns	ns	ns	ns	ns	ns	**<0.001**

**Fig 2:**
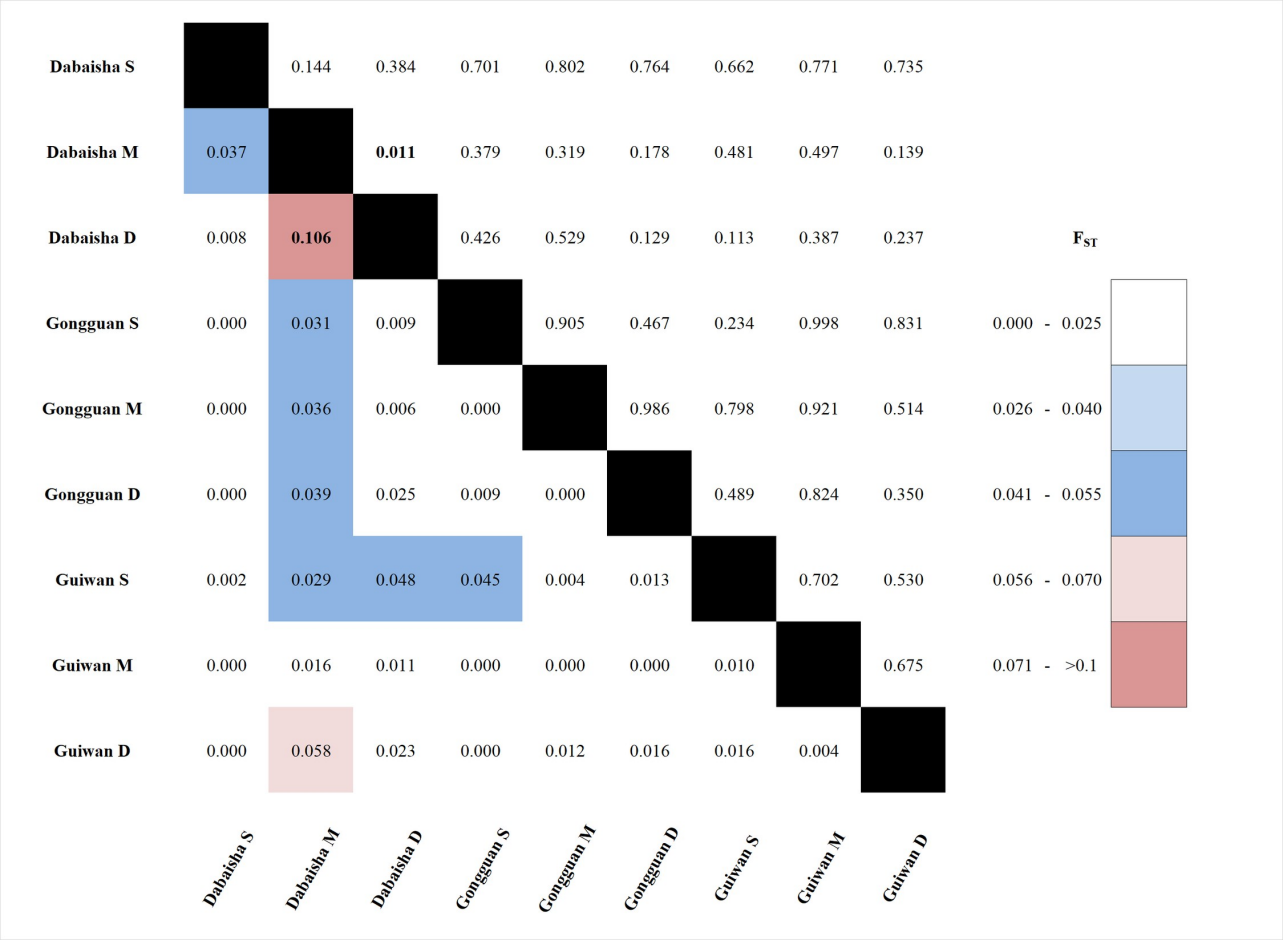
F_ST_ heatmap. F_ST_ (below) with their respective *p*-values (above). Significant values are in bold.

**Fig 3:**
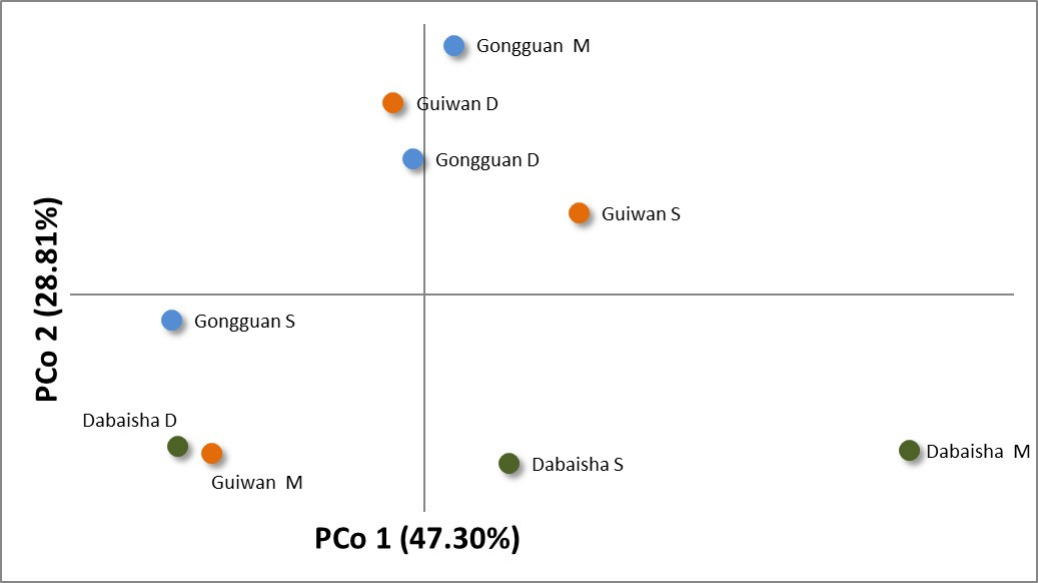
Principal Coordinate Analysis (PCoA) of standardized genotypic distances computed between locations.

Except for the Dabaisha M and Dabaisha D comparison, which displayed significant differentiation (F_ST_ = 0.106, p < 0.05, Fig 2), all F_ST_ values were low (0.000 < F_ST_ < 0.058) and not significant. Pairwise F_ST_ computed at the site and depth levels were also low (0.000 < F_ST_ < 0.007) and non-significant (S1 File). In addition, F_ST_ values that were corrected for the presence of null alleles were not significantly different from non-corrected ones. The PCoA did not reveal any site-specific or depth-related pattern (Fig 3).

Neither STRUCTURE nor CLUMPAK revealed any population structuration (k = 1, Mean LnP(K) = - 1748.400, Prob (k = 1) = 0.688, (Table 3)).

**Table 3:** Estimates and probabilities of K population structure. Mean LnP (K), Ln’ (K), Ln” (K), and Delta K are given using the Evanno method (see [69] for details). Prob (K = k) is the probability for each K given by STRUCTURE. *computed in CLUMPAK.

K	Iteration	Mean LnP(K)	Stdev LnP(K)	Ln’(K)	|Ln’’(K)|	Delta K	Prob(K = k)*
1	3	-1748.400	0.173	-	-	-	0.688
2	3	-1764.700	4.933	-16.300	95.800	19.422	0.000
3	3	-1876.800	47.256	-112.100	102.567	2.170	0.000
4	3	-2091.467	248.563	-214.667	215.633	0.868	0.000
5	3	-2521.767	389.107	-430.300	446.933	1.149	0.000
6	3	-2505.133	632.237	16.633	496.600	0.785	0.000
7	3	-1991.900	375.499	513.233	276.233	0.736	0.000
8	3	-1754.900	0.173	237.000	235.300	1358.505	0.001
9	3	-1753.200	1.453	1.700	1.933	1.331	0.007
10	3	-1753.433	3.329	-0.233	2.300	0.691	0.011
11	3	-1751.367	2.811	2.067	2.333	0.830	0.154
12	3	-1751.633	3.372	-0.267	-	-	0.139

Assignment tests yielded 40% of our specimens (30/75) as potential new migrants: 10 came from deeper locations, 10 came from shallower locations and the remainder relocated from similar depth but from a different site (Table 4). This result was consistent when specimens were grouped by site (10 potential migrants) and depths (10 potential migrants, including 3 detected as coming from shallower locations and 7 detected as coming from deeper locations; S1 File).

**Table 4:** Detection of potential new migrant. For each specimen (Assigned sample) from the location where it was sampled (Origin) the table gives the likelihood (-log (L_home_/L_max_) of this specimen to be a new migrant with its respective probability p < α and the likelihood of originating from another location (-log(L)). Shaded lines highlight potential new migrants their possible location of origin in **bold**. Number of loci (Nb. of loci) are the loci used for calculations (loci with missing data were not included into the calculation).

Assigned sample	Origin	= -LOG(L_home / L_max)	probability	Dabaisha S	Dabaisha M	Dabaisha D	Gongguan S	Gongguan M	Gongguan D	Guiwan S	Guiwan M	Guiwan D	Nb. of loci
				’-log(L)	’-log(L)	’-log(L)	’-log(L)	’-log(L)	’-log(L)	’-log(L)	’-log(L)	’-log(L)	
6001	Dabaisha S	0	0.7121	6.444	9.25	6.721	8.063	7.439	6.573	8.502	6.712	7.792	6
6008	Dabaisha S	1.919	0.0643	13.568	12.477	17.141	18.62	17.363	12.953	11.648	15.916	12.541	8
**6002**	**Dabaisha S**	2.082	0.0499	13.605	14.484	14.433	11.586	**11.523**	12.448	14.395	13.302	11.595	7
**6007**	**Dabaisha S**	2.499	0.0303	8.107	8.472	6.977	7.729	6.688	7.326	**5.608**	7.436	7.201	8
6011	Dabaisha S	0.786	0.2177	9.373	13.268	9.234	8.859	11.171	9.385	8.587	10.466	10.661	8
6018	Dabaisha S	1.411	0.1287	7.651	7.961	8.66	6.24	10.125	7.46	7.648	7.144	7.305	7
6019	Dabaisha S	0.805	0.2186	10.423	13.836	11.058	11.905	9.618	11.324	13.081	11.311	13.247	8
6020	Dabaisha S	0.298	0.3264	8.805	14.359	8.861	8.507	10.28	8.928	11.69	10.056	8.518	8
6021	Dabaisha S	1.765	0.0736	9.487	10.569	8.869	9.178	10.689	7.947	9.327	7.723	8.724	8
6022	Dabaisha S	1.332	0.1239	8.245	7.359	10.991	8.109	6.979	6.913	7.741	7.755	7.895	7
**6024**	**Dabaisha S**	2.53	0.0312	11.126	14.694	**8.596**	11.729	11.643	11.712	10.419	12.527	11.27	7
6026	Dabaisha S	1.117	0.161	11.774	16.903	11.496	12.638	16.384	12.801	12.553	10.658	12.652	8
6027	Dabaisha S	0	0.7021	6.945	9.66	7.672	7.127	8.551	7.017	9.072	7.688	8.122	8
6028	Dabaisha S	1.561	0.0978	7.501	8.915	8.086	8.428	5.94	6.318	7.683	8.047	6.423	8
6032	Dabaisha M	0.299	0.2328	10.389	9.417	16.295	11.285	11.866	9.118	11.737	10.994	9.606	8
6030	Dabaisha M	2.129	0.0768	9.025	9.436	8.961	11.808	9.852	8.892	7.307	11.135	10.456	8
**6031**	**Dabaisha M**	1.929	0.0488	8.036	9.815	8.957	11.234	**7.886**	8.658	10.201	8.527	11.25	6
6033	Dabaisha M	2.116	0.0765	9.128	8.134	12.965	6.018	8.678	8.539	10.52	6.85	7.856	6
**6034**	**Dabaisha M**	4.612	0.0129	11.251	12.63	10.614	11.285	10.572	11.934	**8.018**	11.478	11.699	8
**6035**	**Dabaisha M**	3.001	0.0309	12.1	12.93	13.891	10.257	11.271	10.059	10.909	**9.929**	11.629	8
**6045**	**Dabaisha D**	2.315	0.0437	**7.931**	11.171	10.246	10.428	11.099	10.295	8.225	10.468	12.78	6
6047	Dabaisha D	0	0.6461	10.402	9.949	7.523	7.905	10.359	9.864	10.678	9.079	10.058	6
6048	Dabaisha D	1.476	0.1049	8.824	13.517	10.3	9.638	10.764	10.041	13.006	11.153	12.055	8
**6049**	**Dabaisha D**	2.166	0.0474	7.417	11.66	8.68	6.604	7.639	8.439	7.541	8.767	**6.515**	8
6051	Dabaisha D	0	0.6548	14.964	18.87	13.142	16.939	18.113	15.844	15.389	14.424	16.02	8
6052	Dabaisha D	0.713	0.1674	8.017	10.739	8.379	8.303	8.317	7.913	7.666	8.147	9.502	8
**6053**	**Dabaisha D**	2.183	0.0499	9.409	12.563	9.953	9.377	9.338	**7.77**	9.924	10.181	10.393	8
6054	Dabaisha D	1.02	0.1281	7.791	9.183	7.117	6.604	7.493	6.096	9.316	8.156	7.344	8
6055	Dabaisha D	2.111	0.0576	7.172	11.642	9.082	9.638	9.764	8.275	8.687	8.834	6.97	8
**6060**	**Gongguan S**	5.177	0.0141	11.444	**7.12**	9.869	12.297	11.104	8.941	11.539	8.582	9.548	8
6065	Gongguan S	2.861	0.0721	8.234	10.563	9.769	10.473	10.104	8.051	12.762	7.612	8.201	8
**6067**	**Gongguan S**	3.311	0.0433	7.794	8.517	9.07	9.552	**6.241**	7.114	7.741	6.582	7.775	8
6072	Gongguan S	0.299	0.2958	10.286	10.12	11.058	8.279	9.241	11.001	10.754	7.98	10.578	8
**6073**	**Gongguan S**	7.018	0.0014	8.466	15.012	**7.232**	14.25	8.882	9.71	10.286	10.165	9.162	8
6092	Gongguan M	1.664	0.1271	10.98	12.87	12.109	11.905	10.623	10.168	8.959	13.422	9.456	8
6084	Gongguan M	1.369	0.1506	9.894	12.086	8.246	8.553	8.901	7.532	8.946	10.582	10.046	8
6086	Gongguan M	1.567	0.1284	9.493	12.517	10.517	12.428	10.891	9.323	9.637	10.693	10.474	8
**6082**	**Gongguan M**	6.123	0.003	10.067	12.739	9.116	**8.604**	14.727	11.294	13.13	10.533	12.247	8
**6085**	**Gongguan M**	5.457	0.0079	11.204	11.438	14.45	**7.854**	13.311	9.783	10.535	9.218	11.594	8
**6087**	**Gongguan M**	3.221	0.0404	**9.044**	10.313	11.709	11.933	12.266	9.405	9.741	12.056	9.745	8
**6089**	**Gongguan M**	5.956	0.0051	11.198	13.341	11.654	10.41	14.891	11.376	11.677	12.492	**8.935**	8
**6099**	**Gongguan D**	4.446	0.005	10.104	14.058	13.199	10.683	9.904	12.699	11.436	13.045	**8.254**	8
**6100**	**Gongguan D**	2.489	0.0364	8.298	9.074	11.253	9.984	9.321	9.942	7.683	9.151	**7.453**	8
6094	Gongguan D	0.727	0.1792	9.299	10.614	10.435	8.127	9.493	8.854	8.997	9.387	8.974	8
6095	Gongguan D	1.752	0.0707	7.372	7.563	7.927	8.854	6.378	8.13	7.741	9.084	9.222	8
6096	Gongguan D	0.608	0.2024	10.022	11.04	10.727	10.632	10.308	9.056	9.548	8.448	10.807	8
6097	Gongguan D	1.151	0.1327	8.819	11.086	8.512	11.285	7.873	9.024	9.511	11.862	9.74	8
**6098**	**Gongguan D**	2.237	0.0443	13.16	14.632	12.774	10.78	14.532	12.034	14.653	**9.797**	11.615	8
6103	Gongguan D	0.312	0.2594	10.216	10.773	8.362	10.206	12.007	8.674	12.009	9.135	9.9	8
**6105**	**Gongguan D**	4.134	0.0057	**7.588**	10.183	10.53	10.331	8.998	11.722	13.021	10.299	10.025	8
6101	Gongguan D	0	0.664	9.386	12.347	9.484	8.428	7.564	6.529	7.833	7.807	9.229	7
**3035**	**Guewan S**	7.362	0.0003	13.122	**10.937**	19.766	16.495	18.225	13.745	18.299	14.475	12.713	7
3041	Guewan S	0.735	0.2538	6.694	8.125	7.998	6.16	8.472	7.648	6.896	6.901	7.828	7
**3034**	**Guewan S**	4.217	0.0267	8.811	10.359	11.801	10.41	8.718	**7.405**	11.623	9.679	7.724	8
**3027**	**Guewan S**	2.928	0.0415	**8.639**	9.505	9.885	13.444	10.329	12.1	11.567	10.64	13.222	6
3031	Guewan S	2.978	0.0642	7.201	10.296	7.529	8.206	7.743	7.892	10.179	8.515	7.581	8
3032	Guewan S	2.335	0.0706	7.332	12.824	7.038	8.461	5.969	8.07	8.304	8.34	6.394	6
3033	Guewan S	2.825	0.0831	8.969	11.739	8.709	9.905	6.387	8.895	9.212	10.038	7.566	8
3024	Guewan M	2.956	0.056	8.879	11.484	8.697	9.127	9.25	7.135	9.799	10.091	9.724	8
3016	Guewan M	2.137	0.1076	8.979	12.757	11.45	9.905	11.803	8.699	11.072	10.836	10.168	8
3018	Guewan M	2.673	0.0785	11.157	10.961	10.213	9.729	11.56	9.163	11.799	11.836	10.122	8
**3019**	**Guewan M**	4.327	0.0028	9.391	10.125	**9.225**	9.495	9.356	11.749	11.842	13.553	10.65	5
**3015**	**Guewan M**	3.72	0.0376	9.523	**7.995**	13.463	8.382	10.857	10.629	9.661	11.715	10.907	8
**3021**	**Guewan M**	3.424	0.0446	10.207	13.852	**8.56**	9.433	11.813	9.786	11.741	11.984	11.532	8
3023	Guewan M	2.44	0.0984	8.702	12.727	11.651	9.16	12.056	9.149	8.778	11.142	9.104	8
**3010**	**Guewan D**	3.741	0.0071	11.136	13.62	16.794	13.16	14.858	10.711	**10.611**	13.757	14.352	8
3014	Guewan D	1.597	0.0786	9.877	13.967	14.556	11.433	13.163	12.289	11.755	12.2	11.473	8
**3001**	**Guewan D**	2.238	0.0442	12.161	12.563	12.456	12.507	10.132	**9.904**	9.976	12.181	12.142	8
3002	Guewan D	0.488	0.2171	14.251	17.739	11.852	12.604	14.472	12.539	14.453	13.226	12.339	8
3009	Guewan D	0.557	0.1928	8.349	12.915	8.5	9.382	10.502	9.124	9.507	8.348	8.905	8
3003	Guewan D	0.164	0.2678	9.911	10.53	10.915	10.41	10.28	9.477	11.724	11.755	9.64	8
3004	Guewan D	0.756	0.1831	6.998	9.341	9.135	5.842	8.921	6.868	8.034	7.98	6.598	7
**3005**	**Guewan D**	4.878	0.0016	6.191	9.296	6.131	**5.905**	8.211	7.125	7.842	6.767	10.783	8
**3006**	**Guewan D**	2.82	0.0281	9.042	7.694	10.153	9.331	8.873	8.059	**6.395**	9.302	9.215	7
3008	Guewan D	1.565	0.088	10.637	12.563	9.274	10.03	7.291	8.945	9.862	10.851	8.857	8

## Discussion

*Pocillopora verrucosa* colonies originating from three sites and three depth ranges were genotyped at eight microsatellite loci to evaluate genetic differentiation and genetic structure, and to estimate migration flux between sites and across depths. Overall, *P*. *verrucosa* exhibits a low level of genetic differentiation, with the exception of Dabaisha M and Dabaisha D, which showed a mild genetic differentiation despite being separated by a short horizontal distance. Pairwise F_ST_ values generally support high genetic connectivity between sites and across depths (Fig 2 and S1 File), and the absence of clustering suggested that *P*. *verrucosa* specimens were collected from the same, well-mixed population unit (Table 3). We further identified possible migrants originating from sites/depths different from those where they were collected (Table 4 and S1 File), highlighting that MCEs could act as a source of propagules for nearby shallow water communities (and vice versa). Our data indicate that populations of *P*. *verrucosa* around the oceanic island of Ludao may approximate panmixia, thus we hypothesized that the mesophotic zone around this island could 1) act as an ecological refuge against disturbances for several other depth generalist species inhabiting this area and 2) benefit from the Kuroshio Current for dispersion of coral larvae and increase the overall connectivity along its path.

### Low genetic differentiation and absence of genetic structure across depth in *P*. *verrucosa*

In this study, *P*. *verrucosa* from Ludao exhibited low genetic differentiation between sites located less than 6 km apart, and across depth zonations occurring at shorter horizontal distances. Only one pairwise comparison showed weak but significant differentiation (F_ST_ = 0.106), which corresponded to two adjacent locations at Dabaisha (M: 23–30 m and D: 38–45 m, Fig 3). Scleractinian coral populations may exhibit strong genetic breaks between shallow and mesophotic depths, even when separated by a short horizontal distance (< 1 km) [12, 39, 43, 70]. High levels of genetic differentiation could be linked to biotic factors such as selection against migrants (or genotypes) in their non-native environment [12], temporal differences in reproductive timing [71], and/or depth-dependent parental effects [72]. Alternatively, high levels of genetic differentiation could also be linked to abiotic factors such as contrasting reef geomorphology and water currents between shallow and mesophotic habitats that create a physical barrier to dispersal [39, 70]. In Ludao, there are evidences for shallow and mesophotic reefs to produce recruits in synchrony [73]. For *Pocillopora*, similar molecular operational taxonomic units (MOTU) are recruiting at both shallow and mesophotic depths [73]. In addition, few scleractinian corals from the mesophotic zone have been observed to have gametes, in synchrony with shallow water corals, affirming that these scleractinians are reproductively active (*Fymbriaphyllia ancora*: -45 m and *Acropora* spp.: -40 m, De Palmas S, personal observation). Despite the short horizontal distance separating Dabaisha M from Dabaisha D (< 100 m), both locations are well-known for their strong currents. The slight difference between these two adjacent locations could be due to diverging currents generating a physical barrier to *P*. *verrucosa* larval dispersal. Overall, low genetic differentiation and the absence of any genetic structures within and between sites further suggest that *P*. *verrucosa* specimens originated from a single population, and that this population was probably panmictic.

Previous investigations have found contrasting patterns of connectivity between shallow and mesophotic populations, varying from open to closed populations, with connectivity patterns occurring over thousands of kilometers [39, 43, 70, 74–76]. For instance, populations of the coral *Montastraea cavernosa* showed genetic differentiation with depth in Florida [39] and Little Cayman Island [41], but not in Bermuda or the US Virgin Islands [39, 41]. Similar findings were seen in *Seriatopora hystrix* from the Great Barrier Reef: the species displayed genetic differentiation with depth at Yonge Reef and Day Reef [36], but not at Scott Reef [40]. Interestingly, while it was proposed that broadcast spawners (i.e., species that release gametes into the water column and undergo external fertilization) may have higher dispersal abilities than brooders (i.e., species that release sperm and larvae into the water column and undergo internal fertilization) [36], low genetic differentiation was observed in the broadcasting species *Stephanocoenia intersepta* [12]. In addition, divergence by depth has also been reported in both brooders and spawners [36, 39–41, 43]. *Pocillopora verrucosa* is a broadcasting spawner, which spawns in the early morning [77], 2–3 days after the full moon in April and/or in May in Taiwan [78]. Mulla et al. [78] found larvae of *P*. *verrucosa* to display positive photo-movements, i.e., phototaxis or photokinesis. They further suggested that P. *verrucosa* larvae could disperse further and benefit from stronger surface currents as they move upward into the water column. This larval behaviour could explain the absence of genetic structure observed in the present study. In general, the ability of mesophotic populations to reseed shallow water reefs should be regarded as species- and location-specific [1] with local hydrology playing a key role in disseminating coral larvae [15]. In Ludao, mesophotic and shallow zonations have large scleractinian diversity overlap (37%, [79]), so that many other reef coral species with large bathymetric distribution may contribute to the reproductive refuge.

### Possible new migrants and their origins

Effective ecological connectivity between shallow and deep zonations is achieved under three prerequisite criteria: 1) suitable habitats supporting robust populations, 2) habitats that are physically connected via water or propagule movements, and 3) organisms with life-history traits that enable successful replenishment of both their own populations and those connected to them [45]. In Ludao, Denis et al. [48] and De Palmas et al. [50] reported that *P*. *verrucosa* is one of the dominant species found across the depth gradient. Our data strongly suggest that larval movement creates connectivity between shallow and deep zonations. Our simulation test detected potential migrants between sites and across depths, which reinforces the notion that mesophotic populations can support themselves as well as adjacent populations. About 40% of our specimens were assigned to different populations from where they were collected (Table 3). In this study, loci and sample numbers were relatively low but our methodology produced a similar outcome when individuals were grouped by sites and depths (S1 File). Among the 30 individuals detected as potential migrant, 1/3 were assigned coming from deeper locations, 1/3 were assigned coming from shallower locations and the rest were assigned to similar depth range from different sites. This result suggests strong exchange of individual between sites and depths, showing that both vertical and horizontal larval exchange shape the populations of *P*. *verrucosa* at Ludao. Moreover, this is congruent with the low observed genetic differentiation and absence of population structure between sites and across depths, and suggests that *P*. *verrucosa* disperses effectively around Ludao. Assignment methods based on the analysis of genetic data should still be taken with caution due to the relative limitations induced by the presence of null alleles and the sensitivity of such methods to imperfect data (missing data and/or an unbalanced number of loci) [66]; however, concordant evidence suggests that *P*. *verrucosa* shows effective ecological connectivity around the island. Our study design includes adult colonies that are probably different ages, so the connectivity pattern is likely the result of multi-generational larval exchange, rather than associated with a specific *P*. *verrucosa* cohort. Population genetics comparing adult colonies to newly recruited *P*. *verrucosa* could tackle this issue and allow researchers to evaluate the current populations’ dynamics.

### Deviation from Hardy-Weinberg, linkage disequilibrium, and null alleles

Among all the combinations of sites, depths, and loci, 12 (out of 96) deviated from Hardy-Weinberg equilibrium (Table 1). In addition, linkage disequilibrium—i.e., non-random association of alleles—was found in *Psp_29*/*Psp_39*, and *Psp_32*/*Psp_48*. These markers were not reported to have linkage disequilibrium in *Pocillopora* populations from the Ryukyus for which they were developed [53]. Linkage disequilibrium can be a result of evolutionary forces such as natural selection or genetic drift [80]. However, null alleles could also slightly affect linkage disequilibrium estimators [81]. Microsatellite null alleles occur when flanking regions have accumulated enough mutations to prevent primers from annealing during PCR amplification [62]; they could also be the result of preferential amplification of short alleles (i.e., Short Allele Dominance, SAD) [82]. Consequently, some alleles simply do not amplify during PCR, in which case individuals that are heterozygous will appear to be homozygous following fragment analysis [83]. In this study, five loci (*Psp_29*, *Psp_35*, *Psp_39*, *Psp_41*, and *Psp_48*) were detected as null alleles, but no SAD effect was found at these loci, suggesting that the flanking regions of those microsatellite loci could have accumulated enough variation to prevent marker annealing during PCR. As a deviation from Hardy-Weinberg equilibrium results from a shortage in heterozygotes (or excess in homozygotes), we hypothesize that the high number of potential null alleles found in this study could influence both linkage disequilibrium and deviation from Hardy-Weinberg equilibrium. Null alleles can also influence population differentiation estimators [68], so they require correction for the potential bias they induce, but in our case, no differences were found between biased and corrected F_ST_. Null alleles are common in a large variety of taxa and, interestingly, are often found in taxa with a large population [62]. *P*. *verrucosa* is indeed locally abundant in Ludao, as it is one of the major components of the scleractinian community, residing from very shallow to at least 55 m deep [48, 50]. Population censuses for scleractinian corals inhabiting the mesophotic zone could reveal important elements for evaluating the dynamic of their populations and *in fine* the role of the mesophotic zone as an ecological refuge.

### Cross-specific amplification for *Pocillopora* microsatellite markers

The microsatellite markers used here were developed for a variety of *Pocillopora* species (*P*. *damicornis* [54, 84], *P*. *verrucosa*, *P*. *meandrina* and *P*. *damicornis* [51, 52], *P*. *acuta*, and *P*. *meandrina* [53]), and are known to amplify closely related taxonomic groups [85–87]. However, our analyses found that only eight markers produced both satisfactory amplification and scoring results for *P*. *verrucosa* from Ludao, and several others did not provide any exploitable results (low amplification success, low specificity, Table 1). Cross-specific amplifications of microsatellites among *Pocillopora* species display contrasting results when compared to previous work. Nakajima et al. [53] confirmed that 14 previously described Simple Sequence Repeat (SSR) markers can amplify *Pocillopora* types 1 (*P*. *meandrina* and *P*. *eydouxi*), 3 (*P*. *verrucosa*), 4 (*P*. *damicornis*), 5 (*P*. *acuta*), and 8 (an undescribed *Pocillopora* species), while several other SSR markers could not (*PV3*, *PV5*, *PV6*, *Pd3-002*, *Pd2-003*, *Pd3-010*, and *Pd13*). None of these previously developed markers produced satisfactory results in *P*. *verrucosa* from Ludao. For instance, *PV3*, *PV5*, *PV6*, *Pd3-002*, *Pd2-003*, and *Pd13* had low amplification rates (<50%) and *Pd3-010* did not amplify at all (Table 1). Markers *PV2*, *PV5*, *PV6*, and *PV7* successfully amplified *P*. *verrucosa* specimens from Southeast Africa [88], and most of these markers generally amplify *Pocillopora* species from the Western Indian Ocean, the Southwestern Pacific, Southeast Polynesia [55, 58, 89], and the Red Sea [90]. Among the 13 markers developed by Nakajima et al. [53] for populations from Japan and predicted to amplify most *Pocillopora* species, four (*Psp_1*, *Psp_2*, *Psp_10*, and *Psp_18*) showed no or poor amplification (<25%), and an additional marker (*Psp_33*) was found to have major additional products (Table 1). We hypothesize that the low amplification success of most SSR markers on *P*. *verrucosa* in Ludao, and differences in cross-specific amplification, could be the result of the accumulation of important mutations in the microsatellite flanking regions [91], which may reflect strong differentiation between populations from Taiwan and those for which these markers were developed and tested. Verifying this hypothesis would require sequencing both microsatellite regions and their flanking regions or applying single nucleotide polymorphisms (SNPs), such as used in Taninaka et al. [92], to reveal population difference of *P*. *verrucosa* across large biogeographic scale in the future.

## Conclusions

Our results demonstrate that *P*. *verrucosa* populations around Ludao from 7 m to as deep as 45 m are close to panmictic. This species is more likely to recover from acute disturbances occurring in shallow waters (typhoons or warm water bleaching events) through the recruitment of larvae originating from adjacent shallow and deep habitats. We think that the reproductive status and reseeding capacity of mesophotic corals in this area require further investigations. Ludao, as an oceanic island could act as an important source of recruits along the Kuroshio Current for populations located on its path [93]. However, to the best of our knowledge, no other study addresses genetic connectivity or larval flux between shallow and deep coral populations in Taiwan [48]. In fact, deeper coral ecosystems around Taiwan are generally not the focus of conservation plans [48], and this study calls for greater consideration to be paid to these ecosystems, as they may constitute important reproductive refuge on the path of the Kuroshio current.

## Supporting information

S1 Table Raw microsatellite data at each locus. Data are reported in allele size in bp and missing data are coded 0. Mitochondrial Open Reading Frame (mtORF) haplotype and museum numbers are indicated for each specimen.
(DOCX)

S1 File Pairwise FST comparisons and migration simulation tests at the site (pooling all depth ranges together) and depth (pooling similar depth range from different sites together) levels. (DOCX)
