# Supplementary material for: Strong horizontal and vertical connectivity in the coral Pocillopora verrucosa from Ludao, Taiwan, a small oceanic island
Source: PLoS One. 2021 Oct 11;16(10):e0258181. doi: 10.1371/journal.pone.0258181 (PMC8504772; doi:10.1371/journal.pone.0258181)
Supplement: S1 Table — Data are reported in allele size in bp and missing data are coded 0. Mitochondrial Open Reading Frame (mtORF) haplotype and museum numbers are indicated for each specimen. (DOCX) [file pone.0258181.s001.docx]

**S1 Table. Raw microsatellite data at each locus.** Data are reported in allele size in bp and missing data are coded 0. Specimens were deposited in Academia Sinica Zoological Museum (ASIZC numbers). *See De Palmas et al. for mitochondrial Open Reading Frame haplotype number correspondence in the literature.
