# Supplementary material for: Strong horizontal and vertical connectivity in the coral Pocillopora verrucosa from Ludao, Taiwan, a small oceanic island
Source: PLoS One. 2021 Oct 11;16(10):e0258181. doi: 10.1371/journal.pone.0258181 (PMC8504772; doi:10.1371/journal.pone.0258181)
Supplement: S1 File — (DOCX) [file pone.0258181.s002.docx]

***S1 File***

- ***In order to account for the low sampling number within each of the nine locations (scenario 1, original analyses), pairwise F_ST_ were computed for two additional scenarios (scenarios 2 and 3) as follows:***

*Scenario 1. Nine locations are considered, defined as the combination of two factors:
depths X sites (sample number below, our original analysis)*


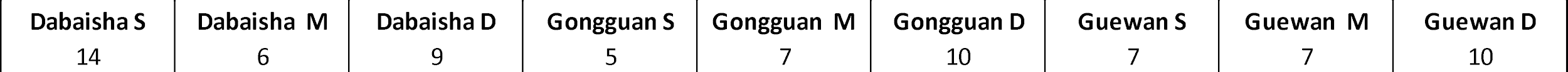


*Scenario 2. Three sites are considered, overlooking the depth factor
(sample number below)*


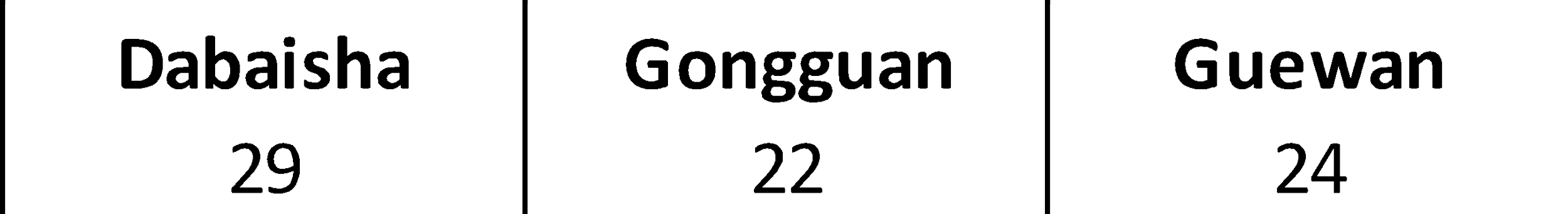


*Scenario 3. Three depths are considered, overlooking the site factor
(sample number below)*


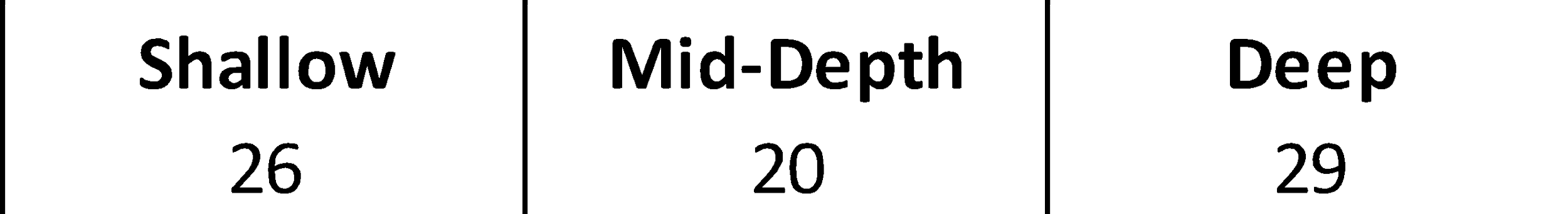


*Here are the F_ST_ values and corresponding p-values for each scenario:*


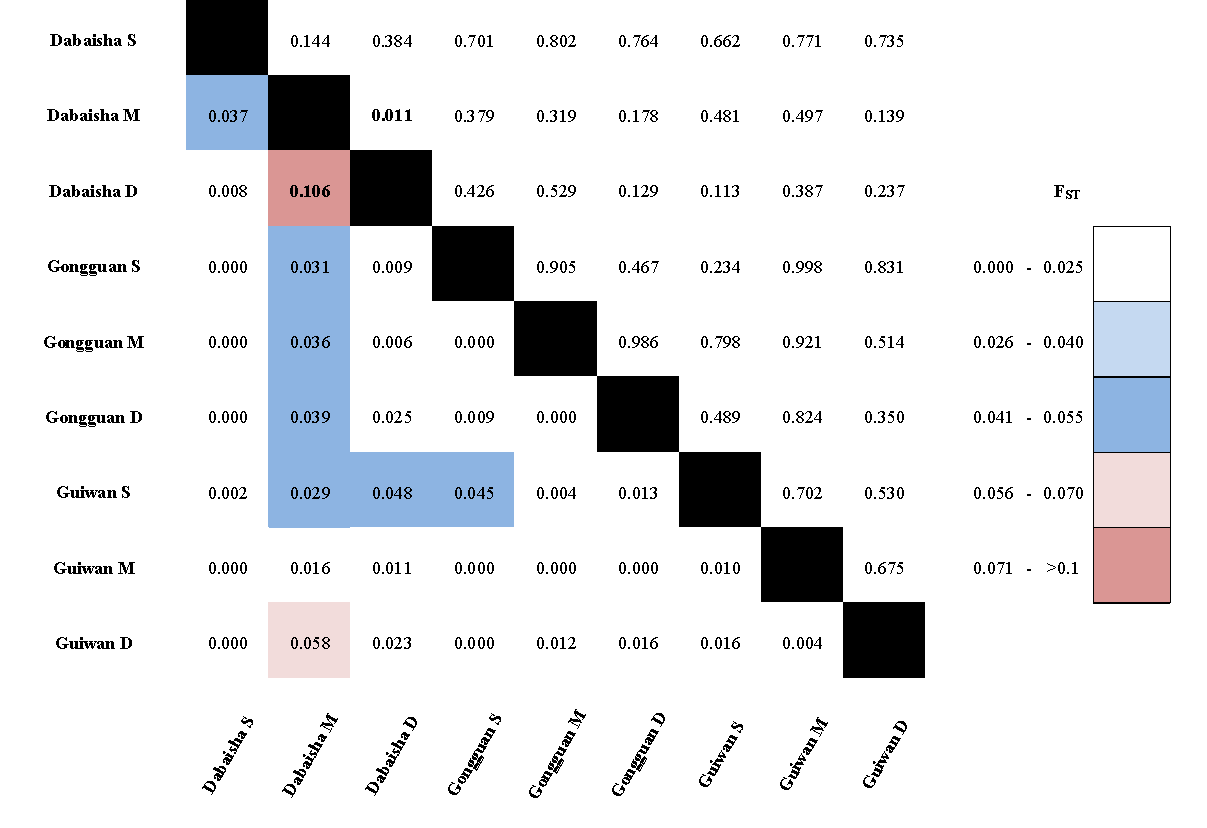


***Fig 1: Scenario 1 F_ST_ heatmap.*** *F_ST_ (below) with their respective p-values (above).* *Significant* *values are in bold.*

*
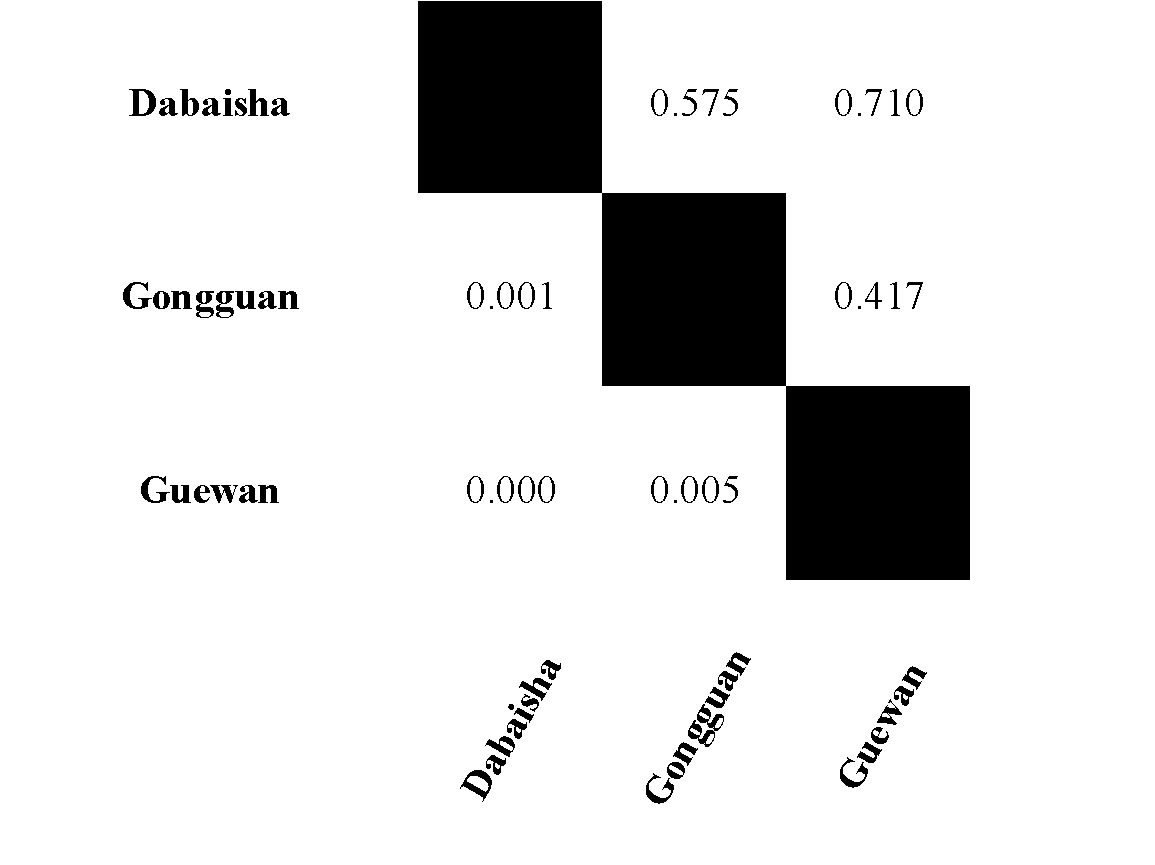

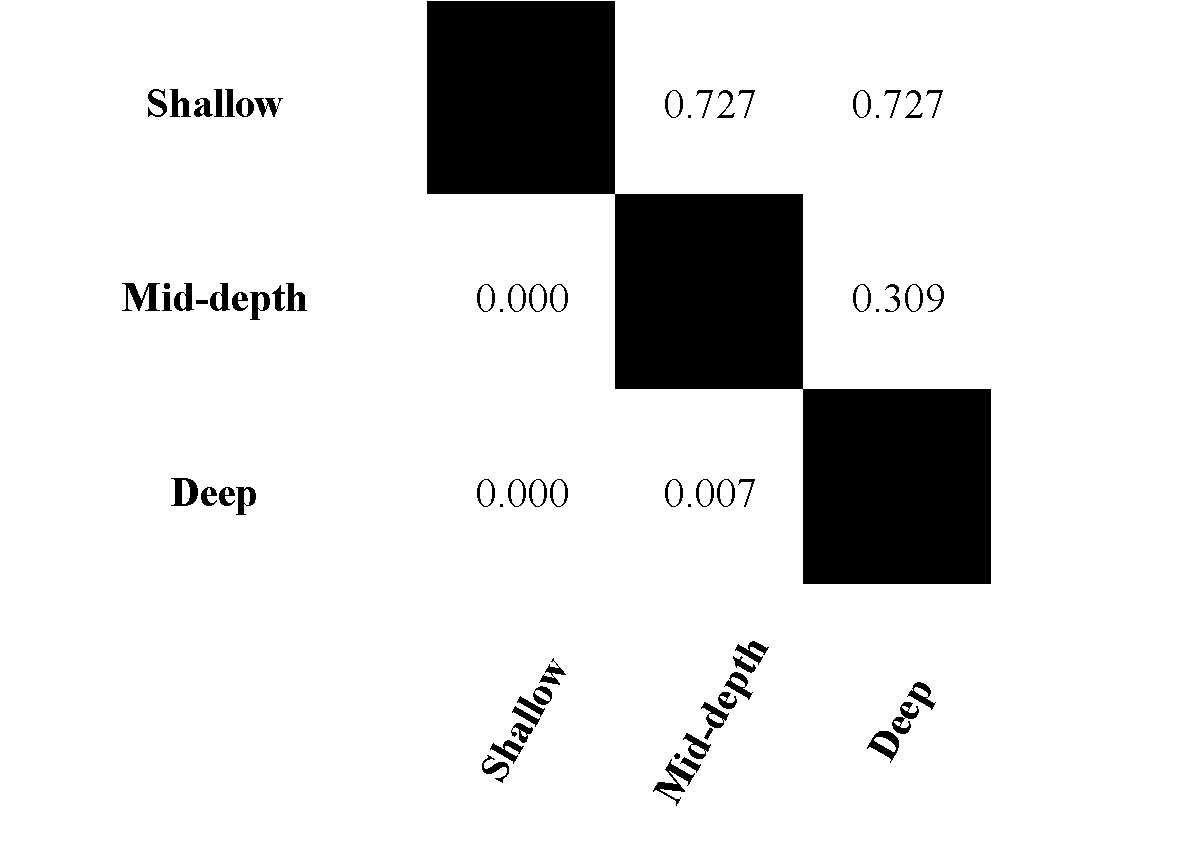
*
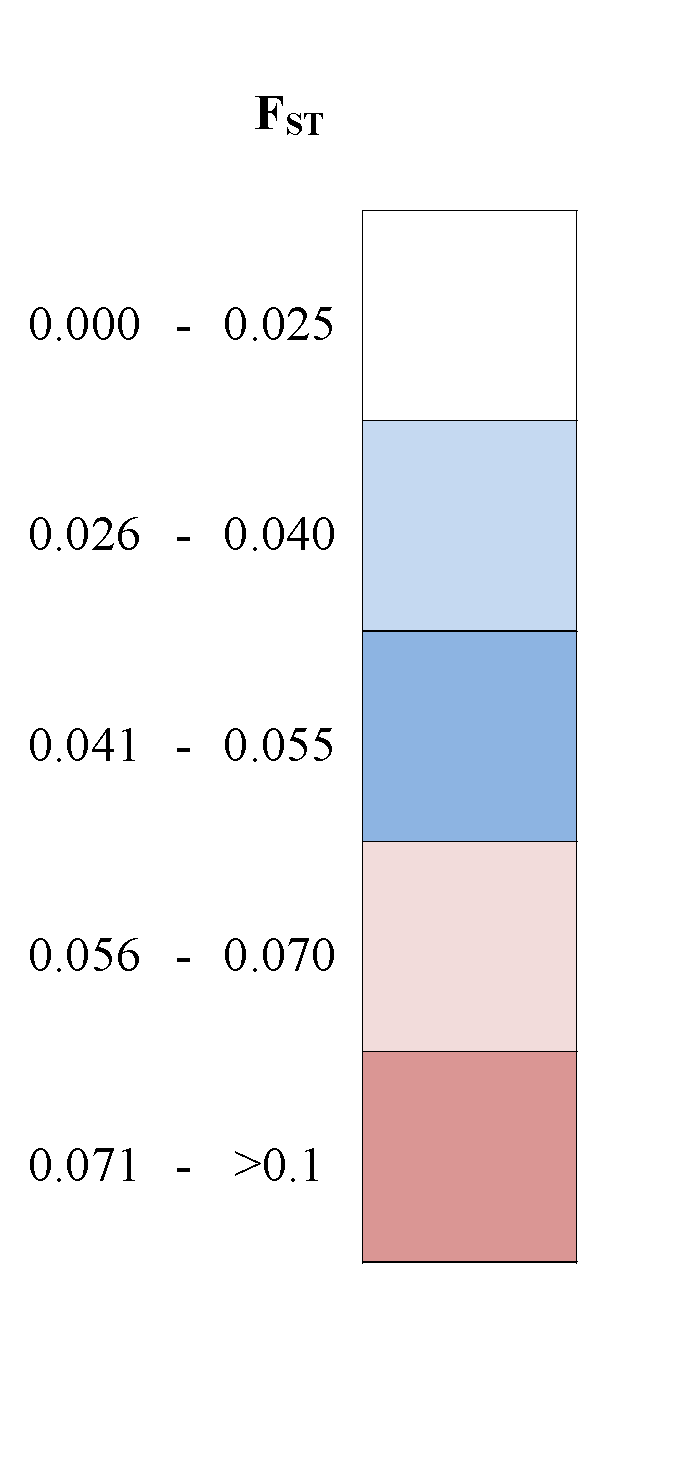


***Fig 2: Scenarios 2 (left) and 3 (right) F_ST_ heatmap.*** *F_ST_ (below) with their respective p-values (above).* *Significant* *values are in bold.*

*Pairwise comparisons between all nine locations (scenario 1) show one weak (F_ST_ = 0.106) but significant (p-value < 0.050) F_ST_ value for Dabaisha M versus Dabaisha D. Overall, differentiation is low and non-significant.*

*In scenarios 2 and 3, pairwise comparisons between sites and depths show very low F_ST_ values (<0.007), all non-significant.*

*Our results show absence of genetic differentiation at the site (scenario 2) or depth (scenario 3) levels, with only a weak signal for differentiation between Dabaisha M and Dabaisha D when both site and depth factors are considered. This suggest that* P. verrucosa *from Ludao could be a panmictic population.*

- ***In order to account for the low sampling number within each of the nine locations (scenario 1), detection of possible new migrant simulation tests was also computed for scenarios 2 and 3.***

*Here are the results of the computation for each scenario:*

***Table 1: Detection of potential new migrant for scenario 1.*** *For each specimen (****Assigned sample****) from the group where it was sampled (****Origin****) the table gives* *the likelihood (****-log (L_home_/L_max_****) of this specimen to be a new migrant with its respective* ***probability*** *p < α and the likelihood of originating from another group (-log(L)). Shaded lines highlight potential new migrants their possible group of origin in* ***bold****. Number of loci (****Nb. of loci****) are the loci used for calculations (loci with missing data were not included into the calculation).*

*Results of scenario 1 simulation highlight 30 specimens (out of 75, 40%) as potential new migrants (collected into one of the 9 groups but originated from another), and show their likelihood of origin (in red). Among them, 10 were detected as coming from deeper populations, 10 were detected as coming from shallower populations and the remainders detected as coming from similar depth but from different sites.*

***Table 2: Detection of potential new migrant for scenario 2.*** *For each specimen (****Assigned sample****) from the group where it was sampled (****Origin****) the table gives* *the likelihood (****-log (L_home_/L_max_****) of this specimen to be a new migrant with its respective* ***probability*** *p < α and the likelihood of originating from another group (-log(L)). Shaded lines highlight potential new migrants their possible group of origin in* ***bold****. Number of loci (****Nb. of loci****) are the loci used for calculations (loci with missing data were not included into the calculation).*

*Results of the scenario 2 simulation highlight 10 specimens (out of 75) as potential new migrants (collected into one of the 3 groups but originated from another), and show their likelihood of origin (in red). Our results show pattern of connectivity between sites, i.e., 2 specimens originally collected at Dabaisha were from Gongguan, 3 specimens originally collected from Gongguan were from Dabaisha, and among 4 specimens originated from Guiwan, half originated from Gongguan and half from Dabaisha.*

***Table 3: Detection of potential new migrant for scenario 3.*** *For each specimen (****Assigned sample****) from the group where it was sampled (****Origin****) the table gives* *the likelihood (****-log (L_home_/L_max_****) of this specimen to be a new migrant with its respective* ***probability*** *p < α and the likelihood of originating from another group (-log(L)). Shaded lines highlight potential new migrants their possible group of origin in* ***bold****. Number of loci (****Nb. of loci****) are the loci used for calculations (loci with missing data were not included into the calculation).*

*Results of the scenario 3 simulation highlight 10 specimens (out of 75) as potential new migrants (collected into one of the 3 groups but originated from another), and show their likelihood of origin (in red). Among them, 3 were detected as coming from deeper populations (collected in Shallow or Mid-Depth but coming from Mid-Depth or Deep) and 7 were detected as coming from shallower populations (collected in Mid-Depth or Deep but originated from Shallow).*

*Overall, these simulations show that* P. verrucosa *are exchanging individuals at the site and depth levels, and further suggest that its population around Ludao is open.*
